# Fighting cardiac fibrosis using the chemomechanical method

**DOI:** 10.1016/j.mbm.2025.100147

**Published:** 2025-08-16

**Authors:** Yunlong Huo

**Affiliations:** Institute of Mechanobiology & Medical Engineering, School of Life Sciences & Biotechnology, Shanghai Jiao Tong University, Shanghai, China

**Keywords:** Diffuse myocardial fibrosis, Matrix softening, TGFβ inhibitor

## Abstract

Diffuse myocardial fibrosis affects disease severity and outcomes in multiple heart diseases. A recent study in NATURE has shown a chemomechanical method to regulate myocardial stromal cell states to suppress fibrosis in vitro and in vivo, which provides a proof-of-concept therapeutic strategy. This study reviews the proposed chemomechanical method and other recent biotechnologies to fight cardiac fibrosis.

Diffuse myocardial fibrosis (DMF) with tremendous clinical impact denotes the diffuse interstitial and perivascular deposition of fibrotic tissues in the myocardium,^1^ which is often encountered in a number of chronic cardiac diseases, e.g., aging, myocardial hypertrophy, and heart failure with preserved ejection fraction (HFpEF). The excessive deposition of collagen fibers through the entire myocardium with DMF increases stiffness of extracellular matrix (ECM) significantly.^2^ This stiffening is a mechanotransduction signal for myocardial fibrogenesis, resulting in a vicious cycle of mechanoactivation and fibrogenesis with time.^3^ Since DMF is proportional to disease severity and outcomes in HFpEF and strategies to therapeutically target DMF remain elusive,^4^ it is of importance to study the mechanical regulation of fibroblast states to suppress DMF.

Cho et al. have recently shown that a combination of biochemical and mechanical signals could regulate myocardial stromal cell states to suppress fibrosis at the cell and tissue levels (Fig. 1a), i.e., dynamic matrix softening coupled with TGFβ inhibitor (TGFβi) demonstrated a substantial decrease in αSMA expression and nuclear YAP1 as well as the loss of αSMA-decorated stress fibers, while these changes were not observed after treatment with TGFβi alone or dynamic matrix softening alone.^3^ This chemomechanical method also triggered a transcriptomic, metabolic and morphological shift of fibroblast activation states towards quiescence in human iPS-cell-derived cells and tissues. In particular, the chemomechanical method disrupted SORBS2 interactions with MRTFA in the nucleus of activated fibroblasts and hence increased its mobility and promoted its exit to the cytoplasm, which could reasonably explain how this method inhibited the MRTF–SRF pathway.

**Fig. 1:**
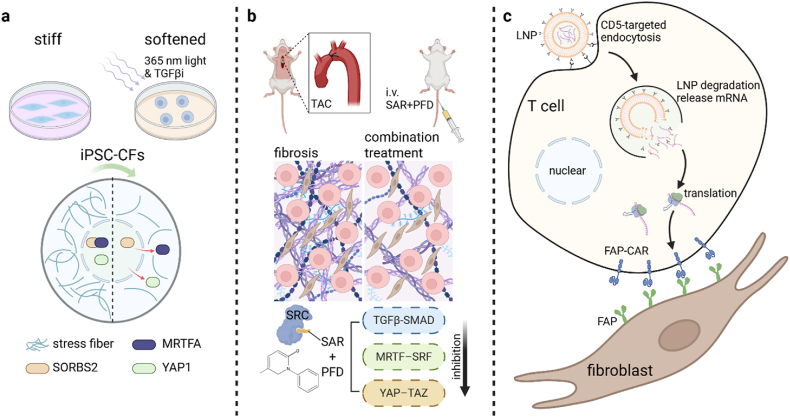
(a) A combination of biochemical and mechanical signals regulating myocardial stromal cell states to suppress fibrosis at the cell and tissue levels; (b) In vivo experiment to test the chemomechanical treatment; and (c) a LNP-mRNA system to transfect T cells in vivo to generate anti-fibrotic transient CAR-T cells.

In the animal model, transverse aortic constriction (TAC) induced a gradual progression of DMF, which was accompanied with a significant increase of the activation state of SRC expression (i.e., a focal-adhesion-associated mechanosensor upstream of the YAP–TAZ pathway).^3^ Cho et al. sought to identify small-molecule inhibitors of SRC by performing a virtual docking screen of >10,000 compounds, which showed saracatinib (SAR; AZD-0530) to be the most promising candidate, capable of accessing SRC's binding pocket with the strongest and most stable interactions. The synergy of SAR and TGFβi (e.g., SAR ​+ ​PFD) inhibited progression of both interstitial and perivascular fibrosis and rescued partial systolic dysfunctions in the TAC mouse heart, while each drug alone had much more modest effects (Fig. 1b). The combination treatment of SAR ​+ ​PFD also induced repression of both key matrix genes and downstream targets of the TGFβ–SMAD, MRTF–SRF and YAP–TAZ pathways.

The chemomechanical treatment showed some promising anti-fibrotic effects and partial functional recovery in the TAC model by suppressing fibroblast activation states, but it didn't work in the mouse model of myocardial infarction (MI).^3^ Activated fibroblasts developed focal fibrosis, resulting in necrosis and loss of myocytes after MI, which was different from DMF.^5^^,^^6^ As the infarcted heart healed between 2 and 8 weeks after the injury,^5^^,^^6^ the number of activated fibroblasts gradually decreased in the infarcted area.^7^^,^^8^ Hence, the chemomechanical treatment was much less effective against MI. Although this treatment provides a proof-of-concept therapeutic strategy, the following studies are still required to assess its safety and potential off-target effects.

On the other hand, CAR-T (chimeric antigen receptor-engineered adoptive T) cells targeting fibroblast activation protein (FAP) have already been used to eliminate activated fibroblasts and reduce myocardial fibrosis.^9^ Since fibroblast activation was only part of a normal wound-healing process in many tissues, the indefinite persistence of engineered CAR-T cells could cause potential myocardial injuries. Furthermore, Rurik et al. developed a therapeutic approach to generate transient anti-fibrotic CAR-T cells in vivo by delivering modified messenger RNA (mRNA) in T cell–targeted lipid nanoparticles (LNPs).^10^ In comparison with the engineered CAR-T cells, the LNP-mRNA method was more attractive for clearance of activated fibroblasts because the transient feature of in vivo CAR-T cells was prone to limiting toxicities and titrate dosing. Recently, we have optimized the LNP-mRNA system to transfect T cells in vivo to generate anti-fibrotic transient CAR-T cells (Fig. 1c).^11^ Biochemical parameters for multiple organs as well as histological analysis showed no injury caused by the LNP-mRNA system. Unlike molecular drugs proposed by Cho et al.^3^ transient FAPCAR-T cells directly cleared activated fibroblasts. The rapid suppression of fibrosis during a short-term time period was an advantage of the LNP-mRNA treatment.

These novel anti-fibrotic treatments raise questions and point to challenges albeit they can benefit patients with DMF. Would these therapies work for severe DMF that can result in less access to activated fibroblasts like solid tumor? Are they easy to produce side effects or drug resistance in patients with DMF? How are they applied to the animal model of MI and MI-induced HF? How do they demonstrate titrate dosing for patients with different degrees of DMF given the supporting role of myocardial collagen fibers in the healthy heart? Hence, future studies are required to repeat these therapies in various animal models in different laboratories and further extend them to clinical trials.

## Ethical approval

This study does not contain any studies with human or animal subjects performed by any of the authors.

## Declaration of competing interest

The authors declared no potential conflicts of interest concerning this article's study, authorship, and/or publication.
